# Effects of meeting steps-based and minutes-based physical activity goals on weight loss in online behavioral weight control: seemingly unrelated regression analysis

**DOI:** 10.1080/21642850.2022.2129654

**Published:** 2022-10-03

**Authors:** Melissa L. Stansbury, Rebecca A. Krukowski, Wen You, Jean R. Harvey, Delia S. West

**Affiliations:** a Department of Exercise Science, Arnold School of Public Health, University of South Carolina, Columbia, SC, United States; b Department of Public Health Sciences, School of Medicine, University of Virginia, Charlottesville, VA, United States; c Department of Nutrition and Food Sciences, College of Agriculture and Life Sciences, University of Vermont, Burlington, VT, United States

**Keywords:** Exercise prescription, obesity, physical activity, interventions, health behavior

## Abstract

**Background:**

Behavioral weight loss programs often prescribe physical activity (PA) goals in terms of minutes/week of moderate-to-vigorous PA (MVPA) and steps/day. However, the impact of meeting each type of goal prescription on weight loss is unclear, particularly in digitally-based (eHealth/mHealth) programs. This secondary analysis of a randomized trial examined the effects of meeting steps-based and minutes-based goals on weight loss in an eHealth behavioral weight control program.

**Methods:**

Adults in the control arm received a 6-month online behavioral weight loss intervention with prescribed weekly goals for daily steps and minutes of MVPA. The number of weeks steps-based and minutes-based goals were met (≥100% and ≥75% thresholds) based on self-reported PA were examined as predictors of 6-month weight loss among those providing weight outcomes (*n* = 172; 81% of control arm) using a systems regression approach.

**Results:**

Participants (BMI 35.6 kg/m^2^; 90.1% female; 48.7 years of age) met weekly goals for MVPA (7.1 ± 6.4 weeks) more often than steps (3.5 ± 5.5 weeks, *P *< .001). Meeting the steps goals (*β* = .24, *P *< .001) and MVPA goals (*β* = .20, *P *< .001) were each statistically significant predictors of weight loss at the 100% threshold; their total effects were not statistically different from one another (*χ*^2^*^ ^*= 1.12, *P *= .29). Similarly, at the 75% threshold for steps goals (*β* = .19, *P *< .001) and MVPA goals (*β* = .19, *P *< .001), each independently predicted weight loss; no differences were detected in their total effects (*χ^2 ^*= .01, *P *= .92). The probability of reaching ≥5% weight loss was comparable between meeting the steps goals and MVPA goals at both adherence thresholds.

**Conclusions:**

Greater attainment of PA goals prescribed as steps and minutes of MVPA independently contribute to similar weight loss outcomes in a 6-month online behavioral weight loss intervention. Future research should determine whether promoting adherence to combined steps-based and minutes-based goals produces better weight loss than utilizing either goal alone and identify strategies that improve adherence.

## Introduction

Physical activity is a key element in standard behavioral treatment of obesity (Jensen et al., 2013), which uses a lifestyle approach to assist individuals with skill development and techniques for behavior change to achieve a healthier weight (Foster et al., 2005). Lifestyle programs, as well as national physical activity guidelines (U.S. Department of Health and Human Services, 2018), have traditionally prescribed exercise goals in terms of minutes of moderate-to-vigorous intensity physical activity (MVPA) (Diabetes Prevention Program Research Group, 2002; Wadden et al., 2006). More recently, steps-based goals (e.g. 10,000 steps/day) have become a popular additional strategy for accumulating physical activity (Creasy et al., 2017; West et al., 2010). This is particularly the case in digitally-delivered, electronic (eHealth) lifestyle programs that utilize connected wearable physical activity trackers. Although the current public health guidelines do not provide explicit daily step recommendations to achieve weight loss (U.S. Department of Health and Human Services, 2018), steps-based goals are appealing due to the simplicity of the metric, contribution of light intensity movement to energy balance, and proliferation of wearable devices to monitor and assess physical activity. Despite the uptake of the combined steps-based and minutes-based goal prescriptions in behavioral weight control programs, the impact of meeting each type of physical activity recommendation on weight loss outcomes has not been fully established. Given that many individuals fall short of the program physical activity targets and adherence deteriorates over time (Acharya et al., 2009; Wing et al., 2004), a deeper understanding about attainment of these prescribed goals is needed to enhance treatment effectiveness and shed light on the optimal physical activity goal prescription in behavioral weight control.

The relationship between adherence to program MVPA recommendations and weight loss has been investigated. For example, meeting MVPA goals in the Diabetes Prevention Program (Wing et al., 2004), as well as in the Look AHEAD study (Wadden et al., 2009), was associated with greater weight losses. Another study reported that individuals who met or exceeded the weekly program MVPA goals lost more weight at 6 months than did those who accrued less than half of the prescribed minutes of MVPA each week (Conroy et al., 2011). However, research is limited regarding whether this association holds for steps-based goals. In a physical activity intervention with a weekly goal of 10,000 steps/day, individuals with overweight who consistently recorded an average of at least 9,500 steps/day lost more weight than those who did not reach this step threshold (Schneider et al., 2006). While this finding suggests a steps-based goal may contribute to weight loss, the study was not conducted within the context of a behavioral weight loss program and did not also include an MVPA goal to examine comparative efficacy of the two different prescriptions. There is also some indication that higher daily step counts are associated with greater weight losses during an in-person behavioral weight loss program that prescribed MVPA goals (Creasy et al., 2018); but again treatment goals for physical activity were only provided in terms of minutes. Another study found that weight losses among individuals randomized to a behavioral lifestyle program that incorporated either a minutes-based physical activity goal or a steps-based goal have been shown to be comparable (Creasy et al., 2017). However, the relative contributions to weight outcomes of each physical activity prescription in a program that combines the two is not known, nor has the impact of the physical activity prescriptions within digitally-delivered weight control programs been explored.

As behavioral treatment for obesity continues to evolve in the digital space without in-person components, it is important to identify how physical activity goal attainment in eHealth programs relates to weight-loss success. Furthermore, consideration of the challenges in promoting physical activity adherence warrants attention to these distinct physical activity prescriptions and the influence they have on treatment engagement. There is sparse research examining steps-based and minutes-based goal prescriptions in behavioral weight control interventions, and this information is critical to guiding obesity treatment structure and optimizing weight management outcomes. Therefore, the purpose of this research was to characterize the effects of meeting steps goals and MVPA goals prescribed concurrently during an online behavioral weight control program on weight loss at 6 months.

## Methods

### Study design

A secondary analysis was conducted of the Internet Assisted Obesity Treatment Enhanced with Financial Incentives (iREACH^3^) study (West et al., 2020), which was a two-arm randomized trial that assessed the effects of financial incentives on weight loss outcomes in an online, group-based behavioral weight control program. A description of the iREACH^3^ study and primary outcomes have been published (West et al., 2020). In brief, eligible individuals were ≥18 years of age with a BMI (kg/m^2^) between 25 and 50, had access to a computer and smartphone with internet, were able to walk for exercise, and were willing to use their personal wearable activity tracker (if they owned one) or a smartphone app of their choice to monitor physical activity. Participants were recruited from two clinical centers and cluster-randomized to either the online group-based behavioral weight loss intervention enhanced by financial incentives (incentivized arm) or to the online group-based behavioral weight loss intervention alone (control arm). All participants gave written informed consent prior to participation in the study. The iREACH^3^ study was approved by the Institutional Review Board at the University of South Carolina and the Committee on Human Research in the Behavioral Sciences at the University of Vermont.

The objective of this sub-study was to evaluate the contributions of meeting the weekly program physical activity goals on weight loss within the context of a standard behavioral weight loss program; therefore, we used de-identified data from the control arm only (*n* = 212) to address the research question. Of the 212 individuals randomized to the control arm, 40 participants were excluded due to missing body weight data at 6 months, leaving a final sample of 172 participants (81%) included in this report.

### Intervention

Participants received an online, group-based behavioral weight loss intervention, which emphasized calorie reduction, increased physical activity, and application of self-regulatory skills, such as goal setting, self-monitoring, and problem solving (West et al., 2020). Text-based, synchronous chats with intact groups of 15–18 participants facilitated by a trained interventionist occurred weekly over the 6 months. Additionally, participants were provided with program goals for weight loss, calorie intake (based on body weight at baseline), and programmatic physical activity. Greater detail of the behavioral intervention has been published previously (West et al., 2020).

Weekly physical activity goals were specified in terms of steps/day and minutes/week of MVPA. All participants started with a prescribed goal of 7,000 steps/day at week 2, which progressed by 1,000 steps every 2 weeks until the steps target reached 10,000 steps/day at week 8. The goal continued at 10,000 steps/day through the remainder of the program. Additionally, all participants received an initial goal of 50 min/week of MVPA at week 3. The MVPA goal increased by 50 min every 2 weeks until the goal of 200 min/week was reached at week 9, and the goal remained at 200 min/week thereafter. All forms of aerobic physical activity (e.g. swimming, biking, etc.) taken at a moderate-to-vigorous intensity counted towards total minutes, although walking at a brisk pace in bouts of at least 10 min was encouraged. Walking is the most commonly reported activity among those successful with weight loss and maintenance (Catenacci et al., 2008) and does not require special skills/equipment.

Participants were instructed to monitor both metrics (i.e. total steps and minutes of MVPA) daily using either a smartphone app (the study recommended MyFitnessPal) or a wearable activity tracker (individuals were invited to use their own personal activity tracker; the study did not provide a device). Participants manually submitted (i.e. self-reported) their total steps and minutes of MVPA on the study website daily. In addition to daily self-monitoring of physical activity, individuals were asked to report their total calorie intake for the day and their body weight. An interventionist emailed participants with individualized feedback and encouragement each week based on submitted records. The feedback addressed the previous week’s physical activity self-monitoring and performance in relation to the program steps and MVPA goals, as well as dietary intake, self-weighing, and attendance at weekly chat group sessions.

### Variables

#### Weight change

Body weight was measured by study staff at baseline and 6 months on a calibrated digital scale to the nearest 0.1 kg. Weight change (dependent variable) from baseline to 6 months was calculated as the percent difference in weight, as well as categorized by attainment of ≥5% weight loss, which is associated with health benefits (Jensen et al., 2013).

#### Adherence to physical activity goals

Adherence variables were defined as (1) the number of weeks [out of 23] an individual met the program steps goal and (2) the number of weeks [out of 22] an individual met the program minutes of MVPA goal, based on physical activity data self-reported on the study website. For each week, individuals were considered to have met the steps goal if their average daily step total across 7 consecutive days was ≥100% of the program goal for that week. Similarly, individuals were classified as having met the MVPA goal if their total self-reported minutes of MVPA for the week was ≥100% of the program target. Adherence to each physical activity goal was also examined based on meeting ≥75% of the program target in a given week, which has been used as a threshold of adherence in other eHealth weight loss programs (Patel et al., 2021). Days with step counts <1,000 and >30,000 (Downs et al., 2009; Rowe et al., 2004) and MVPA >1,080 min/day (Centers for Disease Control and Prevention, 2013; Slagter et al., 2018) were considered implausible values or potential reporting errors and regarded as missing.

### Statistical analysis

Descriptive statistics are presented as mean ± standard deviation (SD) for continuous data or frequency and percentage for categorical data. Differences in baseline characteristics between individuals with weight data at 6 months (‘completers’) and those missing weight data (‘non-completers’) were analyzed using independent *t*-tests for continuous variables and Chi-square or Fisher’s exact tests for categorical variables. SAS version 9.4 (Cary, NC) was used for summary measures and initial analysis.

To investigate the influence of adherence to physical activity goals on weight loss outcomes, we conducted two types of analyses. The first analysis estimated the net effects of each adherence variable, as well as the joint effects, using a single regression model with both adherence variables entered as independent variables. Baseline body weight was included as a covariate given evidence that it is a reliable predictor of weight loss (Wing & Phelan, 2003). The standard errors were adjusted to be cluster-robust to account for group-level clustering in the data. The second analysis estimated the total effects of adherence to each of the physical activity goals through estimating two regression models simultaneously with seemingly unrelated regression (SUR) (Zellner, 1962). Specifically, one model included baseline body weight (covariate) and meeting the steps goals as the independent variables, while the other model included baseline body weight (covariate) and meeting the MVPA goals as the independent variables. This robust system of equations estimation procedure takes into account the potential error term correlation between both equations to provide more efficient estimates of the coefficients and standard errors than is achieved by estimating the equations separately (Anokye & Stamatakis, 2014; Lindner et al., 2020). Furthermore, the correlation matrix of the residuals was examined using the Breusch-Pagan test of independence. The correlation matrix estimated by SUR captures the inter-equation correlation in the standard errors, which enables cross-equation coefficient hypothesis testing (i.e. the statistical differences between the two total effects).

The full analytic approach described was conducted based on adherence to ≥100% of the weekly steps and MVPA goals and then repeated using the 75% threshold for adherence. In addition, analyses at both the 100% and 75% adherence thresholds were conducted with weight loss percentage as the dependent variable. A similar approach was taken to examine categorical weight loss outcomes (≥5% weight loss success vs <5% weight loss) as the dependent binary variable using a systems analysis. In this latter case, we report the marginal effects of meeting the physical activity goals at both the 100% and 75% thresholds for goal achievement on the likelihood of achieving ≥5% weight loss using probit regressions and apply SUR to test the equality of the coefficients. Analyses were conducted using Stata 16.1 (College Station, TX). Statistical significance was set at *P *< .05.

## Results

### Sample characteristics

Demographic details for the study sample (completers) at baseline are shown in Table 1. Participants with objectively measured weight data at baseline and 6 months (*n* = 172, 81.1%) were included in analyses and had a mean BMI of 35.6 ± 5.8 kg/m^2^, 90.1% self-identified as female, and 70.9% self-identified as white. Most had a college degree or higher (83.1%) and were employed full time (83.1%). The analyzed sample (completers only) were significantly older (*P *= .03) and reported higher education (*P *= .01) than non-completers (*n* = 40).

**Table 1.** Baseline demographic characteristics of completers.

Characteristic	*n* = 172
Age (years), mean ± SD	48.7 ± 10.9
Weight (kg), mean ± SD	97.2 ± 19.1
BMI (kg/m^2^), mean ± SD	35.6 ± 5.8
Weight Classification, n (%)	
Overweight	31 (18.0)
Obesity class 1 (BMI of 30 to <35)	57 (33.1)
Obesity class 2 (BMI of 35 to <40)	47 (27.3)
Obesity class 3 (BMI of 40 or higher)	37 (21.5)
Gender, n (%)	
Female	155 (90.1)
Male	17 (9.9)
Race/ethnicity, n (%)	
White^	122 (70.9)
Black	48 (27.9)
Asian	1 (0.6)
Pacific Islander	1 (0.6)
Education, n (%)	
College degree or higher	143 (83.1)
Some college or less	29 (16.9)
Marital status, n (%)	
Married or living as married	97 (56.4)
Single or not married	75 (43.6)
Employment status, n (%)	
Employed full time	143 (83.1)
Employed part time or unemployed	29 (16.9)
Geographic region, n (%)	
Northeast (Vermont)	90 (52.3)
Southeast (South Carolina)	82 (47.7)

^White includes 2 individuals who identified as white and Hispanic; all other races include individuals who identified as non-Hispanic only.

### PA goal attainment and weight change at 6 months

On average, participants met or exceeded 100% of the program minutes of MVPA goals (7.1 ± 6.4 weeks) significantly more often than they met the steps goals (3.5 ± 5.5 weeks; *P *< .001). A similar pattern was found with participants meeting at least 75% of the weekly goals; MVPA goals were met during 9.0 ± 7.1 weeks versus 6.5 ± 7.3 weeks for steps (*P *< .001). Weight loss at 6 months was 5.4% ± 6.1%, on average, with 49.4% of individuals achieving ≥5% weight loss and 19.8% of individuals reaching ≥10% weight loss.

#### Effects of ≥100% PA goal attainment

When examining the effects on weight loss percentage (controlling for baseline body weight), results showed meeting the steps goals while holding MVPA goal attainment constant (*β* = .39, *P *< .013) and meeting the MVPA goals while holding steps goal attainment constant (*β* = .30, *P *< .004) were each statistically significant (i.e. *net effects*). These two net effects were not statistically different from each other (*F*_1,12 _= .23, *P *= .64); however, the combined impact (i.e. *joint effects*) was statistically significant (*β* = .69, *P *< .001).

Results of SUR estimating the *total effects* are shown in Table 2. The model accounted for approximately 20% of the variance in 6-month weight loss percentage and, as expected, there was a high correlation between the equations for steps and MVPA (*r *= .87, *P *< .001). Meeting the steps goal was a significant, positive predictor of weight loss at 6 months, such that each additional week the steps goal was met independently contributed to .24% more weight loss (*z *= 5.35, *P *< .001). Similarly, meeting the minutes of MVPA goal was a significant, positive predictor of weight loss; for each additional week the MVPA goal was achieved, an additional .20% weight loss was expected (*z *= 5.17, *P *< .001). There was not a statistically significant difference between the coefficients (i.e. *total effects*) for meeting steps and MVPA goals (*χ^2 ^*= 1.12, *P *= .29), indicating similar weight losses were predicted when meeting ≥100% of the program-specified steps goal compared to meeting ≥100% of the minutes of MVPA goals.

**Table 2.** SUR estimates of total effects of physical activity goal attainment on weight loss percentage at 6 months.

**STEPS model**								
**Dep. Variable: % weight loss**	**≥100% goal threshold**				**≥75% goal threshold**			
Ind. Variable	*Coeff*	*SE*	*z*	*P*	*Coeff*	*SE*	*z*	*P*
Constant	5.34	1.97	2.71	.007	4.47	1.96	2.28	.022
# of weeks met steps goal	.238	.044	5.35	<.001	.190	.033	5.74	<.001
Baseline body weight	-.008	-.020	-.40	.691	-.003	.020	-.15	.878
	*R*^2^=.203; Root MSE = 5.46; *P** * .001				*R*^2^=.219; Root MSE = 5.40; *P** * .001			
**MVPA model**								
**Dep. Variable: % weight loss**	**≥100% goal threshold**				**≥75% goal threshold**			
Ind. Variable	*Coeff*	*SE*	*z*	*P*	*Coeff*	*SE*	*z*	*P*
Constant	4.05	2.02	2.01	.045	3.47	2.01	1.72	.085
# of weeks met MVPA goal	.200	.039	5.17	<.001	.187	.034	5.48	<.001
Baseline body weight	-.001	.020	-.03	.978	.003	.020	.13	.898
	*R*^2^=.195; Root MSE = 5.49; *P** * .001				*R*^2^=.206; Root MSE = 5.45; *P** * .001			

Note: Based on ≥100% goal threshold, correlation between residuals of STEPS model and MVPA model: .87; Breusch-Pagan test of independence: χ^2^(1) = 130.85, *P *< .001. Based on ≥75% goal threshold, correlation between residuals of STEPS model and MVPA model: .90; Breusch-Pagan test of independence: χ^2^(1) = 138.77, *P *< .001.

When examining weight loss as a binary outcome, the *net effect* of meeting the steps goals at the ≥100% threshold was not statistically significant in predicting ≥5% weight loss (*P *= .17), while meeting the MVPA goals did predict greater likelihood of reaching the ≥5% weight loss threshold (*P *< .001; Table 3). Regarding the *total effects*, the probability of achieving ≥5% weight loss at 6 months significantly increased by 4.5% and 3.8% for each additional week of meeting the steps goal and minutes of MVPA goal, respectively (Table 4). The *total effects* of meeting the steps and MVPA goals were not statistically different from each other (*P *= .91), indicating a similar probability of reaching 5% weight loss was predicted when meeting ≥100% of the program-specified steps goal compared to meeting ≥100% of the minutes of MVPA goals.

**Table 3.** Average marginal effects of meeting steps-based and minutes-based goals on probability of achieving ≥5% weight loss at 6 months using probit regression.

Dep. variable: ≥5% weight loss	≥100% goal threshold				≥75% goal threshold			
Ind. variable	*dy/dx*	*Delta-method SE*	*z*	*P*	*dy/dx*	*Delta-method SE*	*z*	*P*
# of weeks met steps goal	.017	.012	1.36	.174	.017	.008	2.25	.025
# of weeks met MVPA goal	.028	.006	4.40	<.001	.020	.007	3.10	.002
Baseline body weight	-.002	.001	−2.24	.025	-.002	.001	−1.85	.065
	*R*^2^=.236;*χ*^2^(2) = 32.60; *P** * .001				*R*^2^=.276;*χ*^2^(2) = 39.54; *P** * .001

**Table 4.** SUR estimates of total effects of physical activity goal attainment on probability of achieving ≥5% weight loss at 6 months.

**STEPS model**								
**Dep. variable: ≥5% weight loss**	**≥100% goal threshold**				**≥75% goal threshold**			
Ind. variable	*dy/dx*	*Delta-method SE*	*z*	*P*	*dy/dx*	*Delta-method SE*	*z*	*P*
# of weeks met steps goal	.045	.011	4.24	<.001	.035	.004	9.68	<.001
Baseline body weight	-.004	.001	−2.71	.007	-.003	.001	−2.44	.015
	*R*^2^=.178;*χ*^2^(2) = 18.25; *P** * .001				*R*^2^=.241;*χ*^2^(2) = 30.69; *P** * .001			
**MVPA model**								
**Dep. variable: ≥5%****weight loss**	**≥100% goal threshold**				**≥75% goal threshold**			
Ind. variable	*dy/dx*	*Delta-method SE*	*z*	*P*	*dy/dx*	*Delta-method SE*	*z*	*P*
# of weeks met MVPA goal	.038	.003	11.15	<.001	.034	.002	14.49	<.001
Baseline body weight	-.002	.001	−1.81	.070	-.002	.001	−1.45	.146
	*R*^2^=.223;*χ*^2^(2) = 39.05; *P** * .001				*R*^2^=.254;*χ*^2^(2) = 42.63; *P* = < .001			

Note: Test for equality of coefficients in STEPS model versus MVPA model based on ≥100% goal threshold: *P *= .905. Test for equality of coefficients in STEPS model versus MVPA model based on ≥75% goal threshold: *P *= .642.

#### Effects of ≥75% PA goal attainment

A similar pattern was found when considering attainment of ≥75% of the program-specified goals on weight loss percentage, albeit the magnitude of the effects was attenuated. Specifically, the *net effects* of meeting the steps goals while holding MVPA goal attainment constant (*β* = .31, *P *= .004) and meeting the MVPA goals while holding steps goal attainment constant (*β* = .24, *P *= .002) remained statistically significant, and the two *net effects* were not statistically different from each other (*F*_1,12 _= .25, *P *= .62). The *joint effects* remained statistically significant at this lower threshold (*β* = .56, *P *< .001). The *total effects* estimated by SUR (Table 2) indicate that each additional week of meeting ≥75% of the steps goal independently contributed to .19% more weight loss (*z *= 5.74, *P *< .001), while an additional .19% weight loss was expected for every additional week meeting ≥75% of the minutes of MVPA goal was achieved (*z *= 5.48, *P *< .001). No statistically significant difference between the total effects of steps and MVPA on weight loss outcomes at the ≥75% threshold was detected (*χ^2 ^*= .01, *P *= .92).

In terms of the probability in reaching ≥5% weight loss, the *net effect* of meeting the steps goals was statistically significant at the ≥75% goal attainment threshold (*P *= .025), as was meeting ≥75% of the MVPA goals (*P *= .002; Table 3). The probability of reaching ≥5% weight loss at 6 months significantly increased by 3.5% and 3.4% for each additional week of meeting the steps goal and minutes of MVPA goal, respectively (Table 4); however, these two *total effects* were not statistically different from each other (*P *= .64), which indicates the probability of reaching 5% or greater weight loss was comparable between meeting ≥75% of the steps goals and meeting ≥75% of the minutes of MVPA goals.

## Discussion

This study demonstrates that meeting weekly steps-based goals and minutes-based goals prescribed concurrently during online behavioral obesity treatment each offer meaningful contributions to weight loss at 6 months. Findings suggest there are similar weight loss benefits to incorporating either type of physical activity target as part of a comprehensive lifestyle program. The greatest benefits are likely to be achieved when fully meeting either of the prescribed weekly goals for steps or minutes of MVPA; however, reaching 75% of the prescribed goals for steps or minutes of MVPA also appears to confer a meaningful impact on weight loss outcomes, with no difference observed between the two physical activity targets.

To date, research on the associations between meeting program-specified physical activity goals and weight loss during behavioral treatment of obesity have focused on achieving recommendations prescribed in terms of minutes of MVPA with few studies investigating attainment of steps-based targets (Creasy et al., 2017). Not only does the current study reinforce MVPA goal attainment as a reliable positive predictor of weight loss, which is consistent with other reports (Wadden et al., 2009; Wing et al., 2004), it also makes a novel addition to the literature on steps-based recommendations by demonstrating comparable weight losses can be expected through meeting program steps goals. It has been previously reported that weight loss during an 18-month lifestyle program delivered in person can be improved by .21 kg for each additional 1,000 steps/day attained (Creasy et al., 2018). Our results extend this evidence by demonstrating that weight loss is estimated to be enhanced by .24% for each additional week an individual fully meets the program steps goal during the initial 6 months of a digitally-delivered behavioral weight control program. Similarly, a .20% improvement in weight loss is anticipated for every additional week the minutes of MVPA goal is met. Given that both goals appear to impart a comparable magnitude of weight loss, these findings underscore a potential opportunity to personalize treatment and improve adherence by offering individuals a choice between pursuing steps-based or minutes-based physical activity goals.

Although greater attainment of the weekly program physical activity goals was found to predict greater weight loss, it is concerning that steps-based goals were fully met in just 15% of weeks and minutes-based goals were achieved in only 30% of weeks. Previous studies of in-person weight control programs have also described low rates of adherence to physical activity recommendations in lifestyle programs (Acharya et al., 2009; Conroy et al., 2011; Unick et al., 2017), but direct comparisons with the current study are difficult to make due to differences in definitions of adherence and the focus on MVPA goals alone in other studies. Furthermore, the differences in the intervention delivery channel between those studies and the current analysis must be noted. Nevertheless, our findings indicate that steps-based goals were attained in half as many weeks as the minutes-based goals were achieved. This pattern was consistently observed when examining both the 100% and 75% adherence thresholds, suggesting that meeting the prescribed threshold for steps might have been more challenging than meeting the minutes of MVPA recommendations.

This is surprising given research indicating that women engaged in greater physical activity when given goals framed as steps than when provided with minutes of walking goals (Pal et al., 2011). Perhaps the initial threshold and/or the biweekly increases in the steps goals were overly ambitious for the population, particularly considering that adults in the United States take less than 5,000 steps/day, on average (Althoff et al., 2017), which is well below the initial program goal of 7,000 steps/day. A similar discrepancy regarding the MVPA goals may not have existed given reports that adults with overweight/obesity average about 100 min/week (Hooker et al., 2016; Silveira et al., 2022; Young et al., 2009), potentially making the program MVPA goals more achievable. A goal of 10,000 steps/day with 3,500 of those accrued at moderate-to-vigorous intensity has been proposed as the prescription for successful weight control (Creasy et al., 2018); however, it appears that only a select group of individuals reach that level of activity, even by the end of treatment. Our findings that meeting at least 75% of the program steps goal contributed to weight loss success aligns with suggestions by others that 8,000 steps/day may be adequate for weight loss (Tudor-Locke et al., 2008), and thus provides further data to fuel future discussions focused on establishing step-goal recommendations for obesity treatment.

It is also possible that differences in meeting steps-based goals versus minutes-based goals was impacted by modality; the accumulation of steps is constrained to ambulation, whereas MVPA can be accrued through a variety of modalities (e.g. walking, biking, swimming, rowing). Another important consideration is that the program-specified steps goals did not necessarily translate to an equivalent MVPA goal each week. Although, it is important to note the objective of this study was to better understand how adherence to the goals offered in ‘state of the art’ in-person programs, such as the Diabetes Prevention Program (Diabetes Prevention Program Research Group, 2002), relate to weight loss outcomes as opposed to whether the specific goals demonstrate equipoise. Thus, greater exploration of optimal thresholds for steps-based and minutes-based goals for individuals entering a behavioral weight control program are clearly warranted, with particular attention paid to whether uniform program goals for all or individualized physical activity goals reflecting baseline activity level produce the best weight loss outcomes.

It is possible that pursuing multiple physical activity goals may impede a participant’s ability to adhere (Louro et al., 2007; McKee & Ntoumanis, 2014), particularly alongside other behavior change (e.g. calorie reduction) and weight loss goals during treatment. Strategies to increase self-efficacy and persistence towards the goals while reducing temptations that distract from the goals could improve adherence, as these factors have been shown to be associated with successful attainment of multiple goals in the context of weight loss (McKee & Ntoumanis, 2014). Adherence-promotion strategies to produce and sustain increased physical activity, and consequently promote successful long-term weight management, will likely benefit from focusing on both steps-based and minutes-based goals moving forward.

Conceivably, an individual who meets both weekly recommendations might lose more weight than what would be achieved through adhering to a single physical activity goal, but the evidence supporting this hypothesis has not been established. In a pilot randomized trial (Creasy et al., 2017), individuals prescribed a daily step goal demonstrated similar physical activity and weight loss improvements over 3 months to those prescribed a weekly MVPA goal. However, there was not a group that received recommendations for both goals and, thus, this study is silent on whether combined physical activity goals promote greater weight loss relative to singular goals. A more recent study aimed to address this gap by randomizing low-active, older adults to a behavioral weight loss program with physical activity goals prescribed as structured exercise (minutes of MVPA), daily steps, or the combination of minutes-based and steps-based goals, which resulted in comparable weight losses (Fanning et al., 2022). Nevertheless, it cannot be determined whether findings extend to more active and/or younger populations who may have a different response to, or preference for, the two different physical activity goals. Moreover, it remains unclear whether similar outcomes might be observed with minutes-based goals prescribed entirely in a digitally-delivered, free-living, or unsupervised context, since the weight loss program in this study was delivered in person and provided a supervised, structured on-site exercise component during the initial 6 months for those randomized to minutes-based goals. These factors are critical considerations to elucidate whether individuals are most successful in increasing their physical activity and achieving weight-loss success when pursuing one or multiple physical activity goals when delivered remotely.

The following limitations should be considered when interpreting the current study. First, there is a potential for bias due to the use of self-reported physical activity data in these analyses (Adams et al., 2005; Lichtman et al., 1992), which can be mitigated in future studies with objective measures. However, self-reported physical activity tends to be higher than objectively measured activity, suggesting that the goal attainment levels may be even lower than reported here and underscoring the need to emphasize adherence promotion efforts in behavioral weight control. Further, physical activity data were not collected at baseline, which precludes examination of physical activity prior to the start of intervention. In addition, the generalizability of these findings may be restricted given that the sample consisted primarily of college-educated, middle-aged females and analyses were limited to individuals with weight data available at 6 months. This study is also limited to weight loss outcomes at 6 months and may not extrapolate to longer timeframes or weight maintenance, although early physical activity and weight loss are both highly associated with long-term outcomes (Unick et al., 2015; Unick et al., 2020). Given that the SUR model explained only 20% of the variance in weight loss, there are other contributing factors associated with weight loss that were not included in this analysis [e.g. self-monitoring of diet or weight (Burke et al., 2011); weight compensation from increased energy intake and appetite (Martin et al., 2019)] and might interact with the physical activity goals provided. However, other studies have also reported that physical activity accounted for 20% of the variance in weight loss outcomes (Jakicic et al., 2018), aligning with the current study. It is also important to note that despite the high correlation between attainment of the steps-based and minutes-based goals, the net effects found were both highly statistically significant, which minimizes concerns of multicollinearity (Lindner et al., 2020). Finally, a causal relationship between adherence to the program goals and weight loss cannot be determined from these post hoc analyses.

In light of the significant contributions to weight loss outcomes observed for both of the physical activity goals in this exploratory study, future trials examining the effectiveness of single and combined physical activity goal prescriptions on weight loss in a digitally-based behavioral weight control intervention are clearly warranted. In addition, determining how physical activity preferences, motivators, and barriers/facilitators for the two physical activity prescriptions may impact goal attainment, treatment engagement, and ultimate weight loss would substantively advance our understanding of best clinical practices and could offer important guidance for policy statements.

## Conclusions

Findings from this study advance the literature regarding the role of steps-based and minutes-based goals on weight loss in an online behavioral weight control program. The two physical activity goal prescriptions were each independently and similarly associated with weight loss outcomes, suggesting that either type of goal may be recommended as part of a comprehensive behavioral obesity treatment program; however, goal attainment for both steps and minutes was low over the course of the 6-month program. While meeting 100% of the steps-based and minutes-based goals each week may provide the greatest impact on weight loss success, meeting at least 75% of the goals still increased the probability of reaching clinically meaningful weight loss. Therefore, the search for optimal physical activity thresholds for weight loss and behavioral techniques aimed at increasing adherence is critical, while consideration must be paid to whether different strategies may be needed to increase engagement for the two physical activity targets.

## References

[CIT0001] Acharya, S. D., Elci, O. U., & Sereika, S. M. (2009). Adherence to a behavioral weight loss treatment program enhances weight loss and improvements in biomarkers. *Patient Preference and Adherence*, *3*, 151–160. 10.2147/ppa.s5802 19936157 PMC2778406

[CIT0002] Adams, S. A., Matthews, C. E., & Ebbeling, C. B. (2005). The effect of social desirability and social approval on self-reports of physical activity. *American Journal of Epidemiology*, *161*(4), 389–398. 10.1093/aje/kwi054 15692083 PMC2958515

[CIT0003] Althoff, T., Sosič, R., Hicks, J. L., King, A. C., Delp, S. L., & Leskovec, J. (2017). Large-scale physical activity data reveal worldwide activity inequality. *Nature*, *547*(7663), 336–339. 10.1038/nature23018 28693034 PMC5774986

[CIT0004] Anokye, N. K., & Stamatakis, E. (2014). Different conceptual constructs for modelling sedentary behaviour and physical activity: The impact on the correlates of behaviour. *BMC Research Notes*, *7*(1), 921. 10.1186/1756-0500-7-921 25515233 PMC4301058

[CIT0005] Burke, L. E., Wang, J., & Sevick, M. A. (2011). Self-monitoring in weight loss: A systematic review of the literature. *Journal of the American Dietetic Association*, *111*(1), 92–102. 10.1016/j.jada.2010.10.008 21185970 PMC3268700

[CIT0006] Catenacci, V. A., Ogden, L. G., & Stuht, J. (2008). Physical activity patterns in the National Weight Control Registry. *Obesity (Silver Spring)*, *16*(1), 153–161. 10.1038/oby.2007.6 18223628 PMC4578963

[CIT0007] Centers for Disease Control and Prevention. (2013). A data users guide to the BRFSS physical activity questions: How to assess the 2008 physical activity guidelines for Americans. Retrieved June 6, 2021, from https://www.cdc.gov/brfss/pdf/PA%20RotatingCore_BRFSSGuide_508Comp_07252013FINAL.pdf

[CIT0008] Conroy, M. B., Yang, K., & Elci, O. U. (2011). Physical activity self-monitoring and weight loss: 6-month results of the SMART trial. *Medicine & Science in Sports & Exercise*, *43*(8), 1568–1574. 10.1249/MSS.0b013e31820b9395 21200337 PMC4266405

[CIT0009] Creasy, S. A., Lang, W., Tate, D. F., Davis, K. K., & Jakicic, J. M. (2018). Pattern of daily steps is associated with weight loss: Secondary analysis from the step-up randomized trial. *Obesity (Silver Spring)*, *26*, 977–984. 10.1002/oby.22171 29633583 PMC5970037

[CIT0010] Creasy, S. A., Rogers, R. J., Davis, K. K., Gibbs, B. B., Kershaw, E. E., & Jakicic, J. M. (2017). Effects of supervised and unsupervised physical activity programmes for weight loss. *Obesity Science & Practice*, *3*(2), 143–152. 10.1002/osp4.107 28713583 PMC5478811

[CIT0011] Diabetes Prevention Program Research Group. (2002). The Diabetes Prevention Program (DPP): Description of lifestyle intervention. *Diabetes Care*, *25*(12), 2165–2171. 10.2337/diacare.25.12.2165 12453955 PMC1282458

[CIT0012] Downs, D. S., LeMasurier, G. C., & DiNallo, J. M. (2009). Baby steps: Pedometer-determined and self-reported leisure-time exercise behaviors of pregnant women. *Journal of Physical Activity & Health*, *6*(1), 63–72. 10.1123/jpah.6.1.63 19211959

[CIT0013] Fanning, J., Rejeski, W. J., & Leng, I. (2022). Intervening on exercise and daylong movement for weight loss maintenance in older adults: A randomized, clinical trial. *Obesity (Silver Spring)*, *30*(1), 85–95. 10.1002/oby.23318 34932885 PMC8711609

[CIT0014] Foster, G. D., Makris, A. P., & Bailer, B. A. (2005). Behavioral treatment of obesity. *American Journal of Clinical Nutrition*, *82*(1 Suppl), 230S–235S. 10.1093/ajcn/82.1.230S 16002827

[CIT0015] Hooker, S. P., Hutto, B., & Zhu, W. (2016). Accelerometer measured sedentary behavior and physical activity in white and black adults: The REGARDS study. *Journal of Science and Medicine in Sport*, *19*(4), 336–341. 10.1016/j.jsams.2015.04.006 25937313 PMC4609218

[CIT0016] Jakicic, J. M., Rogers, R. J., Davis, K. K., & Collins, K. A. (2018). Role of physical activity and exercise in treating patients with overweight and obesity. *Clinical Chemistry*, *64*(1), 99–107. 10.1373/clinchem.2017.272443 29158251

[CIT0017] Jensen, M. D., Ryan, D. H., & Apovian, C. M. (2013). AHA/ACC/TOS guideline for the management of overweight and obesity in adults. *Circulation*, *129*(25 suppl 2), S102–S138. 10.1161/01.cir.0000437739.71477.ee 24222017 PMC5819889

[CIT0018] Lichtman, S. W., Pisarska, K., & Berman, E. R. (1992). Discrepancy between self-reported and actual caloric intake and exercise in obese subjects. *New England Journal of Medicine*, *327*(27), 1893–1898. 10.1056/nejm199212313272701 1454084

[CIT0019] Lindner, T., Puck, J., & Verbeke, A. (2020). Misconceptions about multicollinearity in international business research: Identification, consequences, and remedies. *Journal of International Business Studies*, *51*(3), 283–298. 10.1057/s41267-019-00257-1

[CIT0020] Louro, M. J., Pieters, R., & Zeelenberg, M. (2007). Dynamics of multiple-goal pursuit. *Journal of Personality and Social Psychology*, *93*(2), 174–193. 10.1037/0022-3514.93.2.174 17645394

[CIT0021] Martin, C. K., Johnson, W. D., & Myers, C. A. (2019). Effect of different doses of supervised exercise on food intake, metabolism, and non-exercise physical activity: The E-MECHANIC randomized controlled trial. *American Journal of Clinical Nutrition*, *110*(3), 583–592. 10.1093/ajcn/nqz054 31172175 PMC6735935

[CIT0022] McKee, H. C., & Ntoumanis, N. (2014). Multiple-goal management: An examination of simultaneous pursuit of a weight-loss goal with another goal. *Journal of Health Psychology*, *19*(9), 1163–1173. 10.1177/1359105313485484 23682068

[CIT0023] Pal, S., Cheng, C., & Ho, S. (2011). The effect of two different health messages on physical activity levels and health in sedentary overweight, middle-aged women. *BMC Public Health*, *11*(1), 204. 10.1186/1471-2458-11-204 21453540 PMC3078883

[CIT0024] Patel, M. L., Wakayama, L. N., & Bennett, G. G. (2021). Self-monitoring via digital health in weight loss interventions: A systematic review among adults with overweight or obesity. *Obesity (Silver Spring)*, *29*(3), 478–499. 10.1002/oby.23088 33624440 PMC12838191

[CIT0025] Rowe, D. A., Mahar, M. I., Raedeke, T. D., & Lore, J. (2004). Measuring physical activity in children with pedometers: Reliability, reactivity, and replacement of missing data. *Pediatric Exercise Science*, *16*(4), 343–354. 10.1123/pes.16.4.343

[CIT0026] Schneider, P. L., Bassett, D. R., Jr., Thompson, D. L., Pronk, N. P., & Bielak, K. M. (2006). Effects of a 10,000 steps per day goal in overweight adults. *American Journal of Health Promotion: AJHP*, *21*(2), 85–89. 10.4278/0890-1171-21.2.85 17152246

[CIT0027] Silveira, E. A., Mendonça, C. R., & Delpino, F. M. (2022). Sedentary behavior, physical inactivity, abdominal obesity and obesity in adults and older adults: A systematic review and meta-analysis. *Clinical Nutrition Espen*, *50*, 63–73. 10.1016/j.clnesp.2022.06.001 35871953

[CIT0028] Slagter, S. N., Corpeleijn, E., & van der Klauw, M. M. (2018). Dietary patterns and physical activity in the metabolically (un)healthy obese: The Dutch Lifelines cohort study. *Nutrition Journal*, *17*(1), 18. 10.1186/s12937-018-0319-0 29433580 PMC5809859

[CIT0029] Tudor-Locke, C., Bassett, D. R., & Rutherford, W. J. (2008). BMI-referenced cut points for pedometer-determined steps per day in adults. *Journal of Physical Activity & Health*, *5*(Suppl 1), S126–139. 10.1123/jpah.5.s1.s126 18364517 PMC2866423

[CIT0030] Unick, J. L., Gaussoin, S. A., & Hill, J. O. (2017). Objectively assessed physical activity and weight loss maintenance among individuals enrolled in a lifestyle intervention. *Obesity (Silver Spring)*, *25*(11), 1903–1909. 10.1002/oby.21971 28940967 PMC5695666

[CIT0031] Unick, J. L., Neiberg, R. H., & Hogan, P. E. (2015). Weight change in the first 2 months of a lifestyle intervention predicts weight changes 8 years later. *Obesity (Silver Spring)*, *23*(7), 1353–1356. 10.1002/oby.21112 26110890 PMC4481874

[CIT0032] Unick, J. L., Walkup, M. P., & Miller, M. E. (2020). Early physical activity adoption predicts longer-term physical activity among individuals inactive at baseline. *Journal of Physical Activity & Health*, *17*(12), 1205–1212. 10.1123/jpah.2020-0114 33152692 PMC9159534

[CIT0033] US Department of Health and Human Services. (2018). *Physical activity guidelines for Americans* (2nd ed.). Washington, DC: US Dept of Health and Human Services.

[CIT0034] Wadden, T. A., West, D. S., & Delahanty, L. (2006). The Look AHEAD study: A description of the lifestyle intervention and the evidence supporting it. *Obesity (Silver Spring)*, *14*(5), 737–752. 10.1038/oby.2006.84 16855180 PMC2613279

[CIT0035] Wadden, T. A., West, D. S., & Neiberg, R. H. (2009). One-year weight losses in the Look AHEAD study: Factors associated with success. *Obesity (Silver Spring)*, *17*(4), 713–722. 10.1038/oby.2008.637 19180071 PMC2690396

[CIT0036] West, D., Berino, J. H., & Krukowski, R. (2010). Long-term outcomes for internet-delivered behavioral obesity treatment. *Obesity*, *18*, S67–S68. 10.1016/j.ypmed.2010.04.018 PMC3101104 20478333

[CIT0037] West, D. S., Krukowski, R. A., & Finkelstein, E. A. (2020). Adding financial incentives to online group-based behavioral weight control: An RCT. *American Journal of Preventive Medicine*, *59*(2), 237–246. 10.1016/j.amepre.2020.03.015 32446752 PMC8510645

[CIT0038] Wing, R. R., Hamman, R. F., & Bray, G. A. (2004). Achieving weight and activity goals among Diabetes Prevention Program lifestyle participants. *Obesity Research*, *12*(9), 1426–1434. 10.1038/oby.2004.179 15483207 PMC2505058

[CIT0039] Wing, R. R., & Phelan, S. (2003). Strategies to improve outcomes and predictors of success. In R. H. Eckel (Ed.), *Obesity: Mechanisms and clinical management* (pp. 415–435). Lippincott, Williams, and Wilkins.

[CIT0040] Young, D. R., Jerome, G. J., Chen, C., Laferriere, D., & Vollmer, W. M. (2009). Patterns of physical activity among overweight and obese adults. *Preventing Chronic Disease*, *62009*, A90. PMC2722396 19527591

[CIT0041] Zellner, A. (1962). An efficient method of estimating seemingly unrelated regressions and tests for aggregation bias. *Journal of the American Statistical Association*, *57*(298), 348–368. 10.1080/01621459.1962.10480664

